# Integrating neural decoding, memristive materials, and adaptive control frameworks for next-generation hippocampal memory prosthetics

**DOI:** 10.1016/j.isci.2026.117268

**Published:** 2026-08-22

**Authors:** Fan Mo, Xiaoyu Zhao, Yuanhong Xu, Chengxuan Tang, Dalin Zhang, Sai Li, Dingyuan Chen, Wenzhi Li, Zhaohui Song, Shaoqi He

**Affiliations:** 1 The Space Information Research Institute, Zhejiang Key Laboratory of Space Information Sensing and Transmission, and College of Materials and Environmental Engineering, Hangzhou Dianzi University, Hangzhou, China; 2 The Third Affiliated Hospital of Wenzhou Medical University, Wenzhou, China; 3 Zhejiang Institute of Artificial Intelligence, Hangzhou, Zhejiang, China

**Keywords:** memory prosthetics, closed-loop brain-computer interface, neural signal processing, neuromorphic materials, memristive devices, bioelectronic interfaces

## Abstract

Memory prosthetics, closed-loop brain-computer interfaces that decode hippocampal activity and deliver adaptive stimulation, are transitioning from animal proof-of-concept to first-in-human trials. Realizing chronically implantable systems requires co-design of three materials-mediated subsystems whose structure-property-processing (SPP) relationships have been treated in isolation: biocompatible electrode interfaces, on-chip neuromorphic computation, and closed-loop control hardware. This review presents an integrated framework. We map neuroscientific findings (theta-phase tracking, theta-gamma coupling, sharp-wave ripple detection) onto engineering specifications for latency, sampling, and charge injection, and onto materials requirements for impedance, switching endurance, and chronic stability. We develop an SPP taxonomy of two dominant materials families: chronic electrode coatings (Pt-Ir, IrOx, PEDOT:PSS, carbon-based, MXene) and oxide memristive synapses (Al2O3/TiO2-x, SrTiO3, HfO2). We further distinguish established findings from emerging directions and flag where small-cohort clinical results have been over-generalized. This synthesis provides materials-design targets for next-generation memory-prosthetic hardware.

## Graphical abstract

**Figure:**
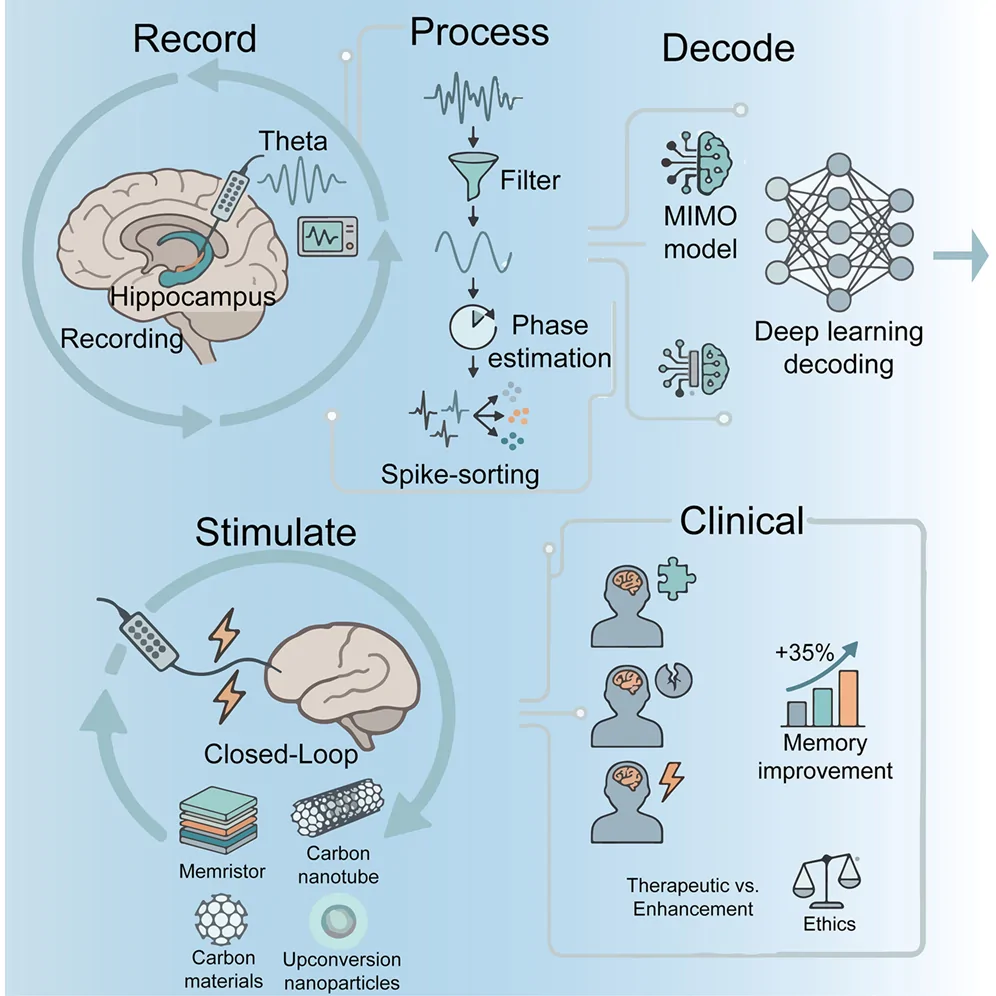

## Teaser

Biotechnology; Bioelectronics; Applied sciences

## Introduction

Memory loss is a major source of disability in Alzheimer’s disease (AD), traumatic brain injury (TBI), and epilepsy, with no pharmacological agent yet shown to restore lost memory function.^1^^,^^2^^,^^3^ Memory prosthetics—closed-loop brain-computer interfaces that decode hippocampal neural activity and deliver patterned stimulation—have emerged as a candidate therapeutic pathway. A first-in-human trial in 8 patients with epilepsy or TBI reported approximately 35% improvement on delayed match-to-sample (DMS) performance using patient-specific MIMO-driven hippocampal stimulation,^4^^,^^5^ providing proof-of-concept; the generality of this effect across larger cohorts, target structures, and task paradigms remains to be established. These advances rest on foundational neuroscience: Tulving’s episodic memory framework,^6^^,^^7^ place cells^8^ and grid cells,^9^^,^^10^ sharp-wave ripples (SWRs) in consolidation,^11^^,^^12^ and recent refinements of entorhinal-hippocampal microcircuitry.^13^^,^^14^ The signal-processing pipelines, decoding algorithms, functional materials, and control architectures required to translate these insights into chronic prosthetic systems remain inadequately synthesized; this review provides such an integration.

A fundamental design contrast underlies the literature. Open-loop high-frequency hippocampal stimulation often impairs memory,^15^^,^^16^ while closed-loop multi-input multi-output (MIMO) models capturing each patient’s encoding dynamics can enhance memory.^4^ Stimulation effects also differ markedly across medial temporal lobe substructures: entorhinal deep brain stimulation (DBS) can rescue memory deficits in mouse AD models,^17^ whereas hippocampal gray-matter stimulation disrupts spatiotemporal memory organization.^16^ These observations frame the engineering problem: where to stimulate (anatomical targeting), when to stimulate (temporal control), and how to maintain adaptive, biocompatible chronic delivery.

**Figure 1:**
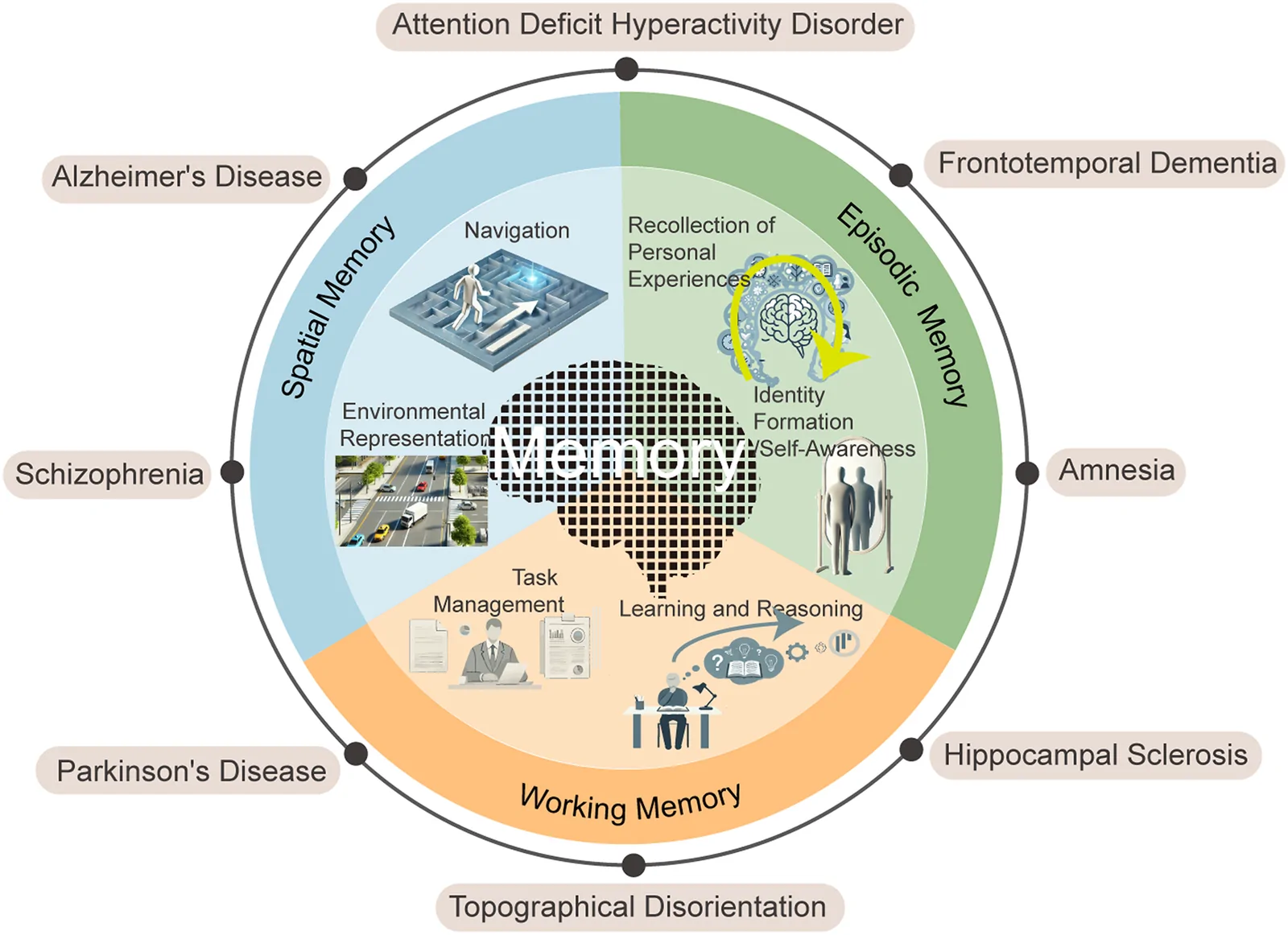
Memory-system impairments across neurological disorders Different conditions show distinct patterns of dysfunction across working, episodic, and spatial memory, providing the foundation for targeted prosthetic design.

Disease heterogeneity adds further constraints (Figure 1). AD primarily manifests as episodic-memory loss with spatial and working-memory deficits,^1^^,^^2^^,^^3^ requiring restoration of hippocampal encoding/consolidation. Schizophrenia shows working-memory deficits in 75–81% of patients,^1^^,^^2^ demanding modulation of prefrontal-hippocampal communication. Frontotemporal dementia (FTD) presents heterogeneous episodic-memory patterns dependent on genetics.^3^^,^^18^ Parkinson’s disease impairs spatial working memory via frontostriatal circuits^19^^,^^20^; ADHD shows executive deficits with dopaminergic dysregulation^21^^,^^22^; hippocampal sclerosis and MTL amnesia produce selective episodic-memory deficits.^23^^,^^24^^,^^25^^,^^26^ This heterogeneity rules out one-size-fits-all designs and motivates patient-specific computational models matched to the disrupted circuit. This review focuses on therapeutic applications—invasive DBS for severe deficits^27^ and non-invasive transcranial temporal interference stimulation (tTIS) for mild-to-moderate impairment^28^—and excludes cognitive enhancement due to lack of ethical consensus.^29^

Two paradigms dominate the prosthetic-design space, and conflating them has been a recurrent source of over-generalization. MIMO-based information-processing operates at the spike-level encoding layer: patient-specific MIMO models translate decoded multichannel CA3 activity into patterned CA1 microstimulation aimed at reconstructing specific information flow.^4^^,^^5^^,^^30^ DBS-style network modulation operates at the field-potential level: macroelectrode stimulation, either continuous high-frequency or phase-locked to ongoing oscillations, biases excitation-inhibition balance and connectivity rather than injecting content.^31^^,^^32^^,^^33^ Clinical evidence is asymmetric: MIMO has been tested in 8 patients,^4^^,^^5^ whereas DBS network modulation has accumulated thousands of patient-years in Parkinson’s disease and dozens of patients in AD trials.^32^^,^^33^ The two paradigms impose different demands on latency, electrode density, personalization, and chronic stability (Table 2; Section closed-loop control architectures: from biomarker detection to adaptive stimulation), and we treat them as complementary layers of a unified architecture rather than competing alternatives.

Hippocampal memory prosthetics sit at the intersection of cognitive neuroscience, signal-processing/control engineering, and materials science of bioelectronic and neuromorphic devices. Terminological barriers have limited cross-disciplinary synthesis: what neuroscientists call “phase precession” maps onto control engineers' “time-varying observable” and onto materials scientists' “sub-25 ms front-end latency with sub-5% conductance variability”. The central thesis of this review is that next-generation memory prosthetics will not be unlocked by single-discipline advances but by the co-design of three materials-mediated subsystems—biocompatible electrode interfaces, on-chip neuromorphic computation, and closed-loop control hardware—whose SPP relationships jointly determine whether decoded neural codes can be delivered with the required latency, fidelity, and longevity. Mankin and Fried^34^ reviewed intracranial memory modulation up to 2020 from a neuroscience and clinical perspective; the present review is differentiated by post-2020 advances,^31^^,^^35^^,^^36^ the addition of a materials/hardware layer, and the formulation of quantitative engineering specifications. Table 1 maps neuroscientific findings to engineering and materials requirements; Sections target selection and temporal control: engineering constraints from neuroanatomy, real-time neural decoding: signal processing pipelines for memory-state detection, and behavioral validation of neural biomarkers: closed-loop performance metrics supply the neuroscience column, Sections closed-loop control architectures: from biomarker detection to adaptive stimulation and computational models and decoding algorithms: from signal processing to adaptive encoding the engineering column, and Sections materials design and hardware implementation: from memristive synaptic devices to neuromorphic platforms and conclusions and future directions the materials column.

**Table 1:** Mapping neuroscientific findings to engineering specifications and materials requirements for hippocampal memory prosthetics

Neuroscientific finding	Engineering specification	Materials requirement
Theta rhythm (4–8 Hz) schedules encoding windows	real-time phase-estimation latency < 25 ms; end-to-end target 10–20 ms	low-impedance coatings (IrOx, PEDOT:PSS); ADC ≥ 2 kSPS; on-chip low-power DSP
HFA (70–140 Hz) marks successful encoding	sampling ≥ 2 kHz; SNR ≥ 30 dB across 70–140 Hz	low-noise interface; PEDOT/IrOx coatings; shielded flexible interconnects
SWRs (80–140 Hz, ∼50 ms) drive offline consolidation	Detection latency < 20 ms; < 5 false positives min^−1^	memristive crossbars for in-memory computing; event-driven ASIC front-ends
Patient-specific CA3→CA1 codes require individualised MIMO models	on-chip weight update; ≥ 16-channel simultaneous record-stimulate	memristive synapses with linear/symmetric LTP/LTD; cycle-to-cycle variability < 5%; endurance > 10^9^
Chronic recording fidelity over months to years	drift compensation; long-term biocompatibility; CIC stability	flexible substrates (polyimide, parylene-C); Pt-Ir/IrOx coatings; graphene with brain-matched modulus

ADC, analog-to-digital converter; CIC, charge-injection capacity; DSP, digital signal processor; HFA, high-frequency activity; SWR, sharp-wave ripple.

## Target selection and temporal control: engineering constraints from neuroanatomy

Section introduction framed prosthetic design as the joint product of neuroscience, engineering, and materials science. This section operationalizes the neuroscience tradition. We trace entorhinal-hippocampal anatomy, oscillatory dynamics, and the spatial/temporal windows that any intervention must respect, deriving the sub-millimeter targeting accuracy, sub-400 μm channel pitch, and charge-injection floors that constrain the materials specifications in Sections closed-loop control architectures: from biomarker detection to adaptive stimulation and materials design and hardware implementation: from Memristive synaptic devices to neuromorphic platforms.

### Biological foundations: Engram cells as targets for materials-mediated intervention

Memory engrams are sparse neuronal ensembles whose causal role has been established by rodent optogenetic activation, silencing, and false-memory experiments.^37^^,^^38^^,^^39^ Retrieval failure rather than memory loss underlies impairments in early AD models, with engram-targeted reactivation restoring access^40^^,^^41^; partial reprogramming of engram neurons via Oct4/Sox2/Klf4 (OSK) gene therapy reverses age-related cognitive decline in mice.^42^ Whole-brain mapping identified ∼117 candidate engram-localized regions whose coordinated reactivation enhances recall,^43^ and CREB-dependent excitability sets the molecular basis of engram allocation.^44^ These findings frame the translational question: how to identify engram-bearing ensembles in humans and reactivate them with the spatiotemporal precision and chronic stability that the Section materials design and hardware implementation: from Memristive synaptic devices to neuromorphic platforms materials platforms must deliver. Because optogenetics is unsuitable for chronic human use, the field converges on multi-site electrical stimulation through high-density biocompatible electrodes. Note that while engram concepts originate in rodent work, the translational target is human declarative memory; we therefore use rodent results as mechanistic constraints rather than as direct evidence for human prosthetics.

**Figure 2:**
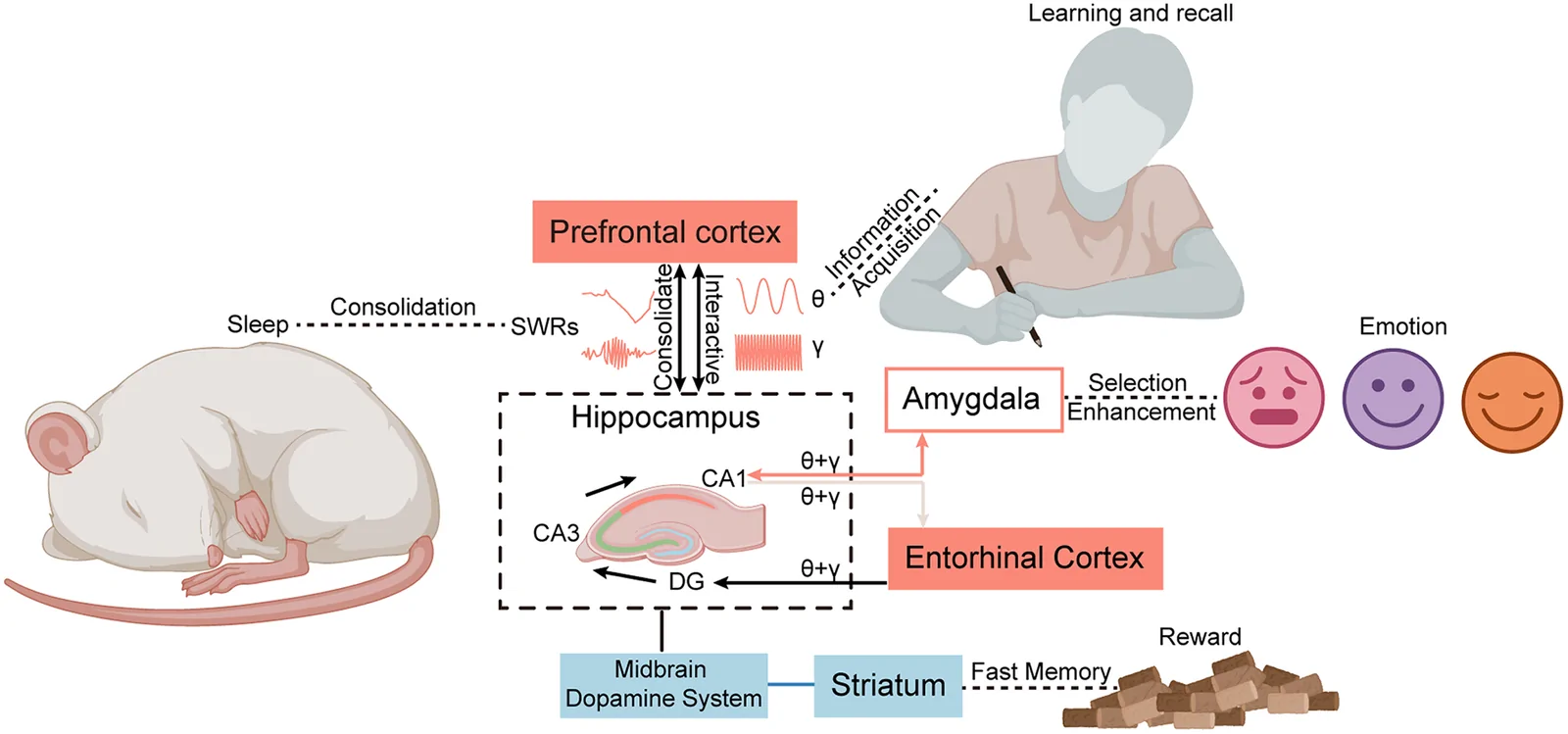
Neural circuits underlying memory formation The hippocampus coordinates with VTA (dopaminergic LTP modulation), PFC (SWR/theta synchrony), amygdala (theta/gamma coupling), EC (distributed storage), and basal forebrain (cholinergic state transitions). These circuit-level constraints translate into engineering targets: VTA dopaminergic timing (0–200 ms post-encoding) defines reinforcement-delivery windows; PFC-hippocampus theta-gamma coupling sets cross-regional synchronization targets; cholinergic transitions signal switching between encoding- and consolidation-enhancement protocols (operationalized in Section closed-loop control architectures: from biomarker detection to adaptive stimulation; materials mapping in Table 1; Section materials design and hardware implementation: from Memristive synaptic devices to neuromorphic platforms).

Effective design requires resolving where and when to stimulate. The hippocampus is the primary target; its encoding, consolidation, and retrieval are governed by theta oscillations (4–8 Hz) providing temporal windows and gamma oscillations (30–150 Hz) representing memory items, with theta-gamma phase-amplitude coupling (PAC) enabling multi-item encoding.^45^ The hippocampus communicates bidirectionally with multiple regions (Figure 2): ventral tegmental area (VTA) dopaminergic projections modulate hippocampal LTP within a 0–200 ms window^46^^,^^47^; prefrontal cortex (PFC) synchronizes via SWRs and theta during working memory and decision-making^48^^,^^49^; amygdala-CA1 theta/gamma coupling (TGC) modulates emotional encoding, with theta-phase differences (1.67 rad) determining encoding success^50^^,^^51^; the entorhinal cortex (EC) hosts grid cells and supports distributed storage.^52^^,^^53^ Recent intracranial recordings show that EC has an enhanced, adaptive role under increased working-memory load, with higher decoding accuracy and cross-regional generalization than hippocampus or lateral temporal cortex (LTC); however, the EC measurements concern working-memory load, an indirect proxy for declarative recall measured in stimulation studies.^35^ The basal forebrain cholinergic system enhances theta synchronization while suppressing SWRs, enabling encoding-consolidation state transitions.^54^^,^^55^ These cross-regional dynamics constrain target selection and stimulation timing (Figure 2).

Anatomical targeting must distinguish white-matter from gray-matter sites with paradigm-specific care. Direct stimulation of human EC and hippocampus impairs memory,^16^^,^^56^ whereas perforant-pathway (angular bundle) theta-burst stimulation has been associated with improved encoding specificity in selected paradigms.^57^^,^^58^ These findings indicate that the superiority of white-matter over gray-matter stimulation is task- and target-specific rather than universal; the cited studies tested different memory operations and cannot be combined into a general rule. Fornix DBS in 42 patients with mild-AD had a negative primary endpoint, with subgroup analysis suggesting age-dependent effects (patients ≥ 65 years potentially benefiting)^32^; connectomic analysis localized the optimal target at the fornix-bed nucleus of the stria terminalis junction, indicating that individual connectivity rather than anatomical coordinates determines efficacy.^33^

Temporal precision is equally critical, but phase-dependent dissociations between encoding and retrieval have not converged on a single principle. In rodent closed-loop optogenetics, Siegle and Wilson reported theta-peak (0°) stimulation improved retrieval by 13.7% and theta-trough (180°) stimulation improved encoding by 10.5%, consistent with CA3-dominated retrieval and EC-dominated encoding^59^; Grennan et al. (2025) extended this peak-versus-trough dissociation to phase-dependent fornix DBS in rodents.^60^ In humans, Kragel et al. delivered theta-trough-locked stimulation to LTC synchronized with hippocampal theta, producing persistent enhancement of network connectivity that was absent under phase-blind controls,^31^ consistent with the “communication through coherence” framework^61^ (analyzed as a case study in Section closed-loop control architectures: from biomarker detection to adaptive stimulation). Whether the rodent peak-versus-trough double dissociation transfers cleanly to humans, and how phase assignments depend on recording reference, target region, and task structure, remain open. Engineering targets in the 10–20 ms range for end-to-end closed-loop latency are typically pursued for reliable theta-phase locking; tolerable latency depends on theta cycle period and required phase precision.^62^^,^^63^

These findings translate into provisional design considerations applicable mainly to encoding-phase modulation of the entorhinal-hippocampal pathway: (1) consider white-matter tracts (perforant pathway) where afferent modulation is intended, rather than treating gray matter as inherently inferior; (2) use theta-band or theta-burst patterns rather than high-frequency continuous stimulation in encoding-related protocols; (3) apply stimulation during encoding or 1–2 s before; (4) target end-to-end loop latency in the 10–20 ms range; (5) base design on patient-specific neural encoding when individual recordings are available. Microelectrodes (∼100 μm diameter) deliver physiological-level current (∼150 μA vs. mA-range for macroelectrodes), reducing non-specific activation. Coating choice (Pt-Ir, IrOx, PEDOT:PSS, carbon nanomaterials) directly influences CIC, impedance stability, and chronic biocompatibility (Section materials design and hardware implementation: from Memristive synaptic devices to neuromorphic platforms).

A particularly relevant hardware advance for hippocampal MIMO prosthetics is the conformal multi-electrode array (cMEA) supporting same-electrode recording and stimulation. Xu et al. demonstrated an early MIMO-compatible cMEA on flexible parylene substrates with electroplated Pt-Ir sites, achieving 16–32 channel coverage of the CA3–CA1 axis in the rodent hippocampus.^64^^,^^65^ Wang et al. extended this with MEMS-fabricated silicon-polymer hybrids with integrated stimulation circuitry.^66^ Elyahoodayan et al. showed that same-electrode record-stimulate operation is feasible without unacceptable artifact contamination when charge-balanced biphasic pulses and blanking windows are co-designed with the front-end amplifier.^67^^,^^68^ These cMEA architectures directly address the channel-count and form-factor requirements imposed by patient-specific MIMO models (Table 1, row 4), and their parylene-C substrates satisfy chronic mechanical-compliance constraints discussed in Section materials design and hardware implementation: from Memristive synaptic devices to neuromorphic platforms.

Implications for materials SPP. Sub-millimeter targeting plus high-channel-count MIMO coverage imposes a processing specification: feature sizes < 50 μm with 100–400 μm channel pitches, requiring photolithography-compatible substrates. The choice of substrate (silicon for high-density penetrating shanks; polyimide or parylene-C for conformal record-stimulate arrays) and coating (electroplated IrOx, PEDOT:PSS, or PEDOT-carbon composites) jointly determines whether the 30–100 μA theta-phase-locked stimulation amplitudes^31^ can be delivered within the Shannon-Pudenz envelope (charge-storage capacity CSC > 4 mC cm^−2^, CIC > 1 mC cm^−2^). White-matter and gray-matter targets impose competing geometric/mechanical demands—stiff penetrating shanks versus brain-modulus-matched compliant cMEAs (≈0.5–2 MPa)—that drive the chronic electrode-materials taxonomy of Section materials design and hardware implementation: from Memristive synaptic devices to neuromorphic platforms.

## Real-time neural decoding: signal processing pipelines for memory-state detection

Section target selection and temporal control: engineering constraints from neuroanatomy established that effective memory modulation requires precise spatiotemporal targeting of the entorhinal-hippocampal circuit. Translating those constraints into a deployable closed-loop system requires real-time identification of memory states, which is the focus of this section. The biomarker definitions, signal-processing pipelines, and front-end electronic requirements must enable implantable systems to detect and act upon memory dynamics within the sub-25 ms latency budget of Table 1. Each biomarker class is traced through to the electrode-coating and integrated-circuit requirements of Section materials design and hardware implementation: from Memristive synaptic devices to neuromorphic platforms.

**Figure 3:**
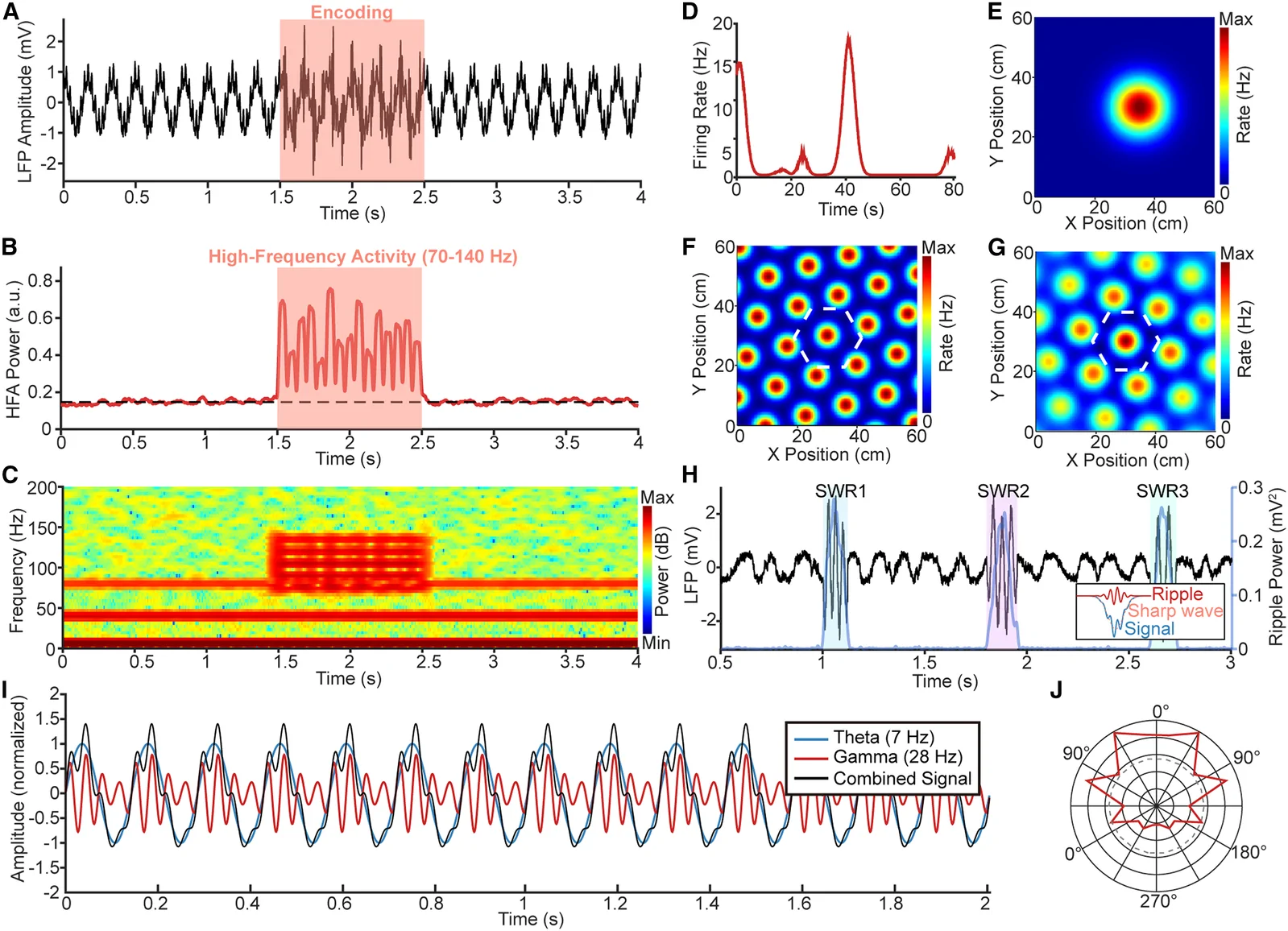
Neural biomarkers for memory-state detection (A–C) HFA (70–140 Hz) as a real-time biomarker of successful encoding: (A) LFP temporal dynamics, (B) power spectral density, and (C) time-frequency spectrogram. (D) Spatial firing-rate map during exploration. (E) Place-cell firing pattern showing spatial selectivity. (F) Grid-cell firing with hexagonal periodicity. (G) Grid-cell spatial autocorrelation revealing hexagonal symmetry. (H) SWRs formed by high-frequency ripple oscillations superimposed on sharp waves. (I) Theta-gamma PAC waveform showing nested oscillations. (J) PAC modulation index (MI) quantification. These biomarker classes impose distinct hardware requirements: HFA and SWR detection require ≥ 1–2 kHz sampling, single-unit decoding ≥ 30 kHz, and phase-locked stimulation total-loop latencies of 10–20 ms (Table 1). These requirements directly constrain the electrode material, front-end-amplifier, and on-chip processing choices developed in Section materials design and hardware implementation: from Memristive synaptic devices to neuromorphic platforms. The figures themselves anchor the design discussion in Section materials design and hardware implementation: from Memristive synaptic devices to neuromorphic platforms: HFA and PAC measurements set ADC and front-end-noise specifications; SWR detection determines the event-driven ASIC architecture; spatial coding patterns motivate ≥ 16-channel record-stimulate cMEAs.

The closed-loop paradigm of NeuroPace RNS in epilepsy provides proof-of-concept: continuous intracranial-EEG monitoring detects seizure-onset patterns and triggers responsive stimulation with 40–60% median seizure reduction.^69^^,^^70^^,^^71^ Memory-system biomarkers are more complex and distributed, requiring simultaneous monitoring across multiple frequency bands and regions. High-frequency activity (HFA, 70–140 Hz) is selectively enhanced during successful encoding^72^^,^^73^ (Figures 3A–3C). In 148 patients with intracranial EEG, hippocampal/amygdala HFA increased during successful emotional encoding, and 50-Hz direct stimulation reduced HFA and impaired memory, providing causal evidence.^15^ HFA detection requires intracranial electrodes (ECoG or depth) because scalp EEG is volume-conduction-limited at these frequencies.^74^

Beyond frequency-domain features, spatiotemporal population structure provides informative biomarkers. Rodent studies established that hippocampal place cells and entorhinal grid cells build a spatial scaffold for episodic memory (Figures 3D–3G),^8^^,^^9^^,^^10^^,^^75^ with predictive grid-cell scanning^76^ and forward/reverse sequence replay.^77^ Although first defined in rodents, the core computational principles (spatial coding, sequence compression, theta phase precession) have been validated in human entorhinal-hippocampal microelectrode recordings^78^; we note, however, that rodent place/grid coding is most relevant as a mechanistic constraint, with declarative-memory decoding being the primary translational target. Capturing structured population activity imposes minima used throughout this review: ≥ 16 simultaneous channels, Bayesian or time-lagged-correlation decoders, and sub-second latency from spike capture to decoded output. Reliable biomarkers require both behavioral correlation and causal manipulation. Fernández-Ruiz et al. showed that longer ripples (>100 ms) correlate with better memory (Figure 3H); optogenetic extension improved working memory while late-phase termination impaired it.^79^ Yang et al. demonstrated that awake SWRs “tag” specific trials for consolidation,^80^ suggesting that detecting and enhancing specific (rather than all) ripples requires content-decoding capabilities.

Biomarker detection imposes hardware constraints: HFA/SWR monitoring requires ≥ 1 kHz (ideally ≥ 2 kHz) sampling; single-neuron classification ≥ 30 kHz for sub-millisecond timestamps; phase estimation requires high-quality LFP and < 20 ms processing latency. Spatial coverage requires bilateral hippocampus plus prefrontal/temporal cortex. The signal-processing pipeline includes online filtering, phase estimation (Hilbert transform), real-time PAC/MI computation, and artifact suppression. Computational, hardware, and materials challenges include cross-regional high-rate acquisition, broadband-recording power, long-term biomarker drift, and electrode degradation. Priorities are integrated low-power neural-interface chips with on-chip signal processing, advanced electrode materials with chronic stability, drift-adaptive online-learning calibration, and unsupervised deep-learning pattern recognition across timescales.

### Design principles for real-time decoding

Six provisional rules follow: (1) capture HFA (70–140 Hz) with intracranial electrodes at ≥ 2 kSPS per channel; (2) detect SWRs with end-to-end latency < 20 ms and false-positive rate < 5 min^−1^; (3) estimate theta phase in real time with < 20 ms processing latency, favoring zero-phase IIR or on-chip Hilbert implementations; (4) combine spectral biomarkers with population decoders (≥ 16 channels; Bayesian or time-lagged correlation) to capture sequence/place content rather than scalar power alone; (5) integrate stimulation-artefact blanking and adaptive drift compensation at the front end; (6) co-design the recording front end with an event-driven, in-memory computing back end to keep instantaneous power within the ∼100 mW thermal budget. These rules apply primarily to invasive depth- or ECoG-based architectures; non-invasive scalp- or temporal-interference systems differ in latency floor and SNR in kind rather than degree.

### Implications for materials SPP

The < 25 ms total-system latency for theta-phase locking imposes ADC sampling ≥ 2 kSPS per channel, on-chip Hilbert or zero-phase IIR filtering, and event-driven digital logic within a < 100 mW thermal budget. At the device level, this collapses into a requirement for in-memory computing: multiply-accumulate operations must execute without crossing the memory-logic boundary that consumes 60–80% of energy in conventional pipelines. The only mature substrate satisfying the area/latency/power triplet is the memristive crossbar, whose analog non-volatile conductance collapses MAC into a single Ohm-plus-Kirchhoff step. Reliability depends on tight processing specifications (cycle-to-cycle variability < ∼5%, retention > 10^5^ s, endurance > 10^9^ cycles), translating into deposition-thickness, anneal, and electrode-stack constraints developed in Section materials design and hardware implementation: from Memristive synaptic devices to neuromorphic platforms.

## Behavioral validation of neural biomarkers: closed-loop performance metrics

Behavioral validation bridges candidate biomarkers and clinically meaningful endpoints. Each biomarker class maps onto a corresponding behavioral readout—primacy effects for encoding efficiency, path-efficiency and decision-point latency for spatial memory, temporal-order judgments for sequence memory—and must be assessed against evidence standards (effect size, cohort size, evidence tier) before claims of clinical utility are warranted.

The primacy effect provides a standardized metric: preferential recall of sequence-initial items reflects enhanced hippocampal-prefrontal synchronization during early encoding.^81^^,^^82^ Tulving’s episodic-memory framework emphasized temporal-spatial context^7^^,^^82^; selective impairment of primacy effects indicates disrupted hippocampal processes. Successful theta phase-locked stimulation should strengthen encoding advantages for sequence-initial items^83^; parietal-montage theta stimulation has also been shown to enhance semantic-memory consolidation.^84^ Spatial biomarkers connect directly to place- and grid-cell mechanisms. In Suthana et al.'s spatial learning task, EC stimulation produced shorter paths and faster speeds, quantified as reduced path length and navigation errors,^58^ with effects observed only on stimulated trials. Left-right alternating theta scanning provides a neural mechanism for spatial prediction,^76^ potentially explaining the effectiveness of EC stimulation for prospective navigation. Path efficiency, decision-point latency, and spatial working-memory span should therefore be included as outcomes.

Temporal-order memory yields a third biomarker class. High-frequency hippocampal stimulation particularly impairs temporal organization, with increased sequence-order errors but preserved item recognition,^16^ supporting the hippocampus’s role in binding temporal information. Buzsáki’s space-time theory emphasizes theta-sequence compression of behavioral-scale trajectories into 100–200 ms cycles.^11^^,^^85^^,^^86^ Phase precession provides the cellular mechanism: neurons fire at progressively earlier theta phases as place fields are traversed, sequentially activating place-cell ensembles within single cycles.^78^ Dual-task paradigms developed for sensorimotor prosthetics also transfer: invasive sensory feedback maintained spelling accuracy during walking with gait speed increasing by 0.103 m s^−1^^87^; non-invasive feedback showed comparable benefits (0.139 m s^−1^) without improved embodiment,^88^ suggesting cognitive offloading can occur independently of perceptual quality.

Behavioral biomarkers should satisfy: (1) cross-individual reliability; (2) convergent validity with neurophysiology (e.g., primacy correlating with TGC); (3) sensitivity to experimental manipulation; (4) clinical significance; and (5) practicality. Standardized batteries should include DMS for short-term memory, delayed recognition (DR) for long-term retention, virtual Morris water maze, and episodic-memory assessments. Limitations of existing measurements—insufficient ecological validity, inability of short-term assessments to predict long-term benefit, and possible divergence of mechanism from design objective due to neural reorganization—motivate ecological momentary assessment, multidimensional composite endpoints, and computational deconstruction of multiple neural contributions.

## Closed-loop control architectures: from biomarker detection to adaptive stimulation

Hippocampal memory-prosthetic strategies fall into two complementary paradigms differing in intervention layer, control objective, and personalization demand. Table 2 makes this distinction concrete. MIMO-based information-processing and DBS-style network-modulation systems are not interchangeable: they operate on different neural variables (spike sequences vs. field-potential oscillations), pursue different control goals (content reconstruction vs. excitation-inhibition rebalancing), and stand at different stages of clinical translation. The architectures and pipelines discussed below apply to both paradigms, with paradigm-specific weighting: MIMO imposes stricter requirements on multi-channel single-unit yield and patient-specific model training; DBS-style systems prioritize long-term lead stability and chronic stimulation safety.

**Table 2:** Conceptual comparison of MIMO-based information-processing and DBS-based network-modulation paradigms in hippocampal memory prosthetics

Dimension	MIMO-based information processing	DBS-based network modulation
Intervention layer	neural encoding (spike-level)	network rhythms (field-potential level)
Control objective	reconstruct specific information flow (e.g., CA3→CA1 successful-trial pattern)	modulate excitation-inhibition balance and network connectivity
Input feature	CA3 multi-channel spike trains; patient-specific MIMO output	LFP band power and phase (theta 4–8 Hz, gamma 30–90 Hz); TGC, SWR rate
Output form	patterned spatiotemporal pulse sequences via microelectrode array	periodic or phase-locked pulse trains via macroelectrode or DBS lead
Patient specificity	high: individualized model trained on each patient’s “successful trials”	moderate: parameter sweep over frequency, amplitude, pulse width, optionally phase
Current clinical evidence	first-in-human trial in 8 patients with epilepsy/TBI^4^^,^^5^; theta-trough-locked LTC stimulation in 14 patients^31^	thousands of patient-years in Parkinson’s; fornix DBS in 42 patients with mild-AD^32^^,^^33^
Inherent limitations	requires high-yield multi-unit recordings and sufficient successful-encoding trials; chronic model drift	cannot deliver specific memory content; effects largely state-dependent rather than information-specific
Primary materials demand	high-density microelectrode arrays (PEDOT:PSS, IrOx, carbon); on-chip MIMO/neuromorphic computation	chronically stable macroelectrode leads; high-CIC coatings; low-latency phase-detection front-ends

The two paradigms differ in intervention layer, control objective, neural input, stimulation output, patient specificity, current clinical evidence, and inherent limitations. They are treated throughout as complementary layers of a unified prosthetic architecture rather than competing alternatives.

**Figure 4:**
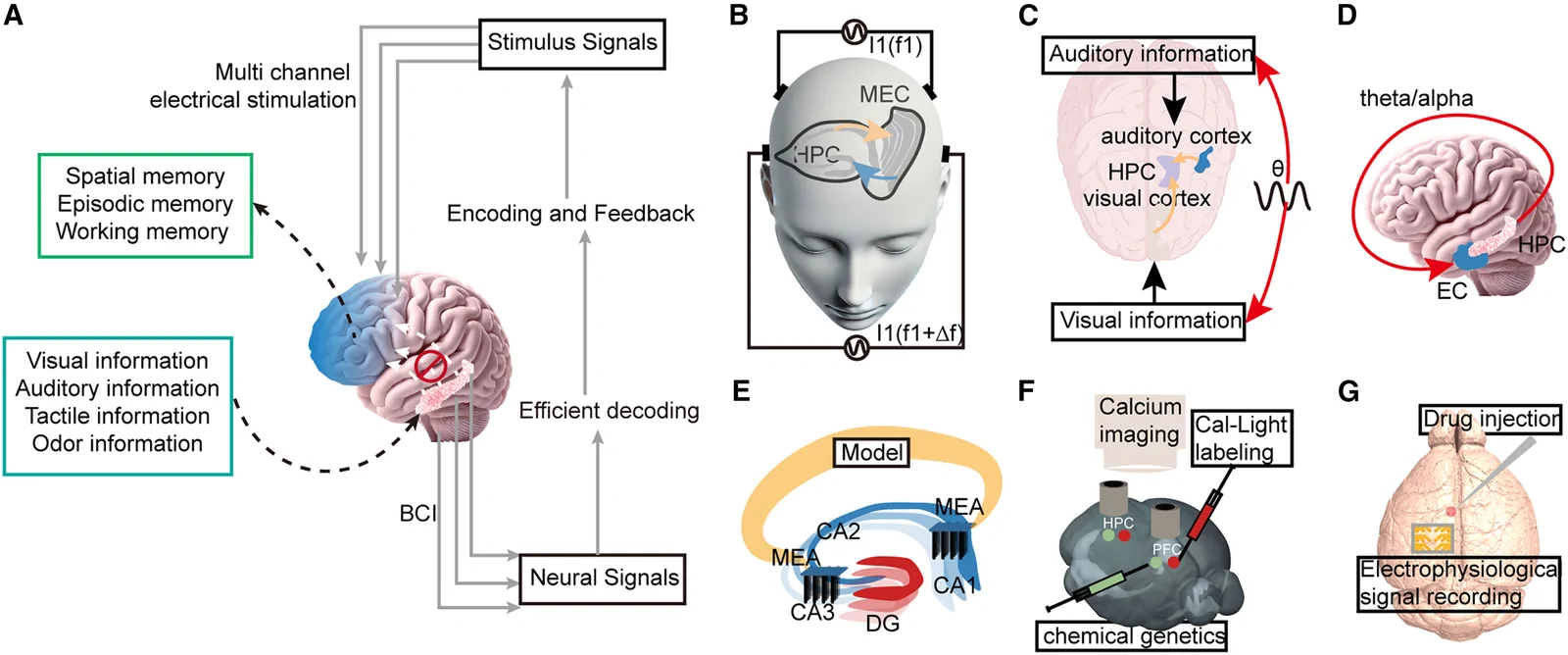
Closed-loop neural prostheses (A) Principles and workflow. Non-invasive: (B) transcranial temporal interference electrical stimulation; (C, D) multi-region tACS targeting specific areas. Invasive: (E) hippocampal-targeted prosthetics with integrated detection-modulation arrays; (F) optogenetic stimulation combined with calcium imaging and electrical modulation; and (G) pharmacological stimulation integrated with microelectrode-array detection.

Closed-loop neuromodulation is the most advanced control architecture: stimulation parameters are dynamically adjusted based on real-time neural-state estimation. Functionally, memory prosthetics integrate detection and stimulation through closed-loop design (Figure 4A): after sensory input, hippocampal signals are recorded by microelectrode arrays, decoded and re-encoded into stimulation patterns, and delivered to the cortex. TGC has been highlighted as a candidate real-time indicator of memory-system state. Daume et al. showed that hippocampal theta-gamma PAC indicates working-memory load (stronger at load 1 than load 3), with PAC neurons shaping population code geometry through noise correlations and exhibiting enhanced cross-regional theta synchronization with ventromedial PFC as cognitive demand increases.^89^ PAC strength negatively predicts reaction time and predicts correct vs. error trials. These correlations have motivated proposals to use TGC as a candidate state variable for closed-loop control.^89^ However, current evidence is correlational; direct demonstrations that PAC-triggered closed-loop stimulation outperforms phase-blind or fixed-schedule control remain to be established.

TGC detection uses MI from phase-amplitude relationships, with z-scoring against trial-shuffled surrogates^90^^,^^91^ (Figures 3I and 3J). Invasive depth and ECoG provide optimal signal quality; scalp EEG/MEG can detect TGC at frontal midline theta sites but with reduced spatial resolution.^92^ In 14 patients with epilepsy, gamma (28 Hz) was modulated by theta (7 Hz) during Sternberg working-memory tasks, with coupling strength predicting capacity.^93^ Cross-regional PAC (xPAC) between hippocampal theta and cortical gamma predicts encoding success, strongest in posterior hippocampus, with EC and parahippocampal cortices showing the strongest xPAC.^94^

SWRs are a core biomarker of hippocampal offline consolidation.^11^ They are highly synchronized population events of tens of thousands of neurons, predominantly during offline states, appearing as 40–100 ms large-amplitude negative deflections in CA1 stratum radiatum, driven by CA3 burst inputs.^95^^,^^96^ Functional roles include compressed replay (5–20×),^11^^,^^12^ memory tagging, bidirectional replay, and systems consolidation. Closed-loop SWR manipulation has been validated across paradigms: post-learning SWR truncation impairs spatial-memory consolidation^97^; awake SWR disruption impairs spatial working-memory learning^98^; optogenetic suppression decreases spatial place-field stability^99^; SWR truncation weakens spatial/social memory while SWR extension enhances working memory.^100^ Counter-evidence exists: Deceuninck et al. found no immediate impairment after awake-SWR truncation in repeated-acquisition tasks, suggesting awake SWRs operate on long-term timescales.^101^ Online SWR detection and closed-loop fornix interruption have been demonstrated in non-human primates.^62^ RMS-threshold detection (150–200 Hz band) achieves 97% true positives at 2.48 false positives min^−1^, with 10–20 ms latency acceptable.^62^^,^^102^

SWR detection requires cross-species standardization: human SWR frequency (80–140 Hz) is slower than rodent, requiring high-pass filtering > 80 Hz, *Z* score normalization, and 15–150 ms duration thresholds,^95^ while distinguishing SWRs from epileptiform activity.^103^ Enhancement strategies include triggering cortical stimulation on SWR onset to strengthen consolidation^104^ and selective interruption of unwanted-memory SWRs^105^; content-decoding identifies replayed trajectories from population activity, enabling selective modulation.^106^^,^^107^ Closed-loop SWR manipulation has been proposed as a speculative direction for trauma- and anxiety-related conditions such as generalized anxiety disorder (GAD) and post-traumatic stress disorder (PTSD),^108^^,^^109^ but clinical translation here is at a formative stage, and no direct evidence of SWR-targeted closed-loop therapy in these patient populations has yet been reported.

Theta power itself is a reliable indicator of memory-circuit engagement, generated by medial-septum/diagonal-band inputs and local hippocampal circuits with multiple distributed generators.^110^^,^^111^^,^^112^ Functions include encoding (active learning/exploration),^110^^,^^113^ sequence compression,^113^ phase precession,^111^ and temporal coding.^114^ Goyal et al. distinguished functionally distinct human hippocampal theta: high-frequency (8 Hz) in the posterior hippocampus (spatial navigation, speed modulation) and low-frequency (3 Hz) in the anterior hippocampus (non-spatial cognition).^112^

Building on the role of theta power as a real-time index of memory-circuit engagement, Kragel et al. (2025) provided the first causal demonstration that theta-phase-locked stimulation can persistently alter human hippocampal network connectivity.^31^ In 14 patients with epilepsy, the system continuously monitored hippocampal CA1 LFP, predicted upcoming theta troughs in real time, and delivered single biphasic pulses to the LTC phase-locked to the predicted trough. Compared with a phase-blind, charge-matched control, the theta-trough-locked protocol increased hippocampal theta power and LTC-hippocampus theta-phase synchrony during stimulation, and elevated stimulation-evoked-potential amplitudes and resting hippocampal-temporal connectivity after stimulation ended, consistent with a plasticity-like after-effect. This persistent change in network connectivity is qualitatively distinct from the acute encoding-phase enhancement reported for entorhinal-area stimulation.^58^ The cohort size (*n* = 14, Tier III, Table 3) precludes population-level generalization; nevertheless, the protocol establishes hippocampal-theta-locked closed-loop stimulation, targeting the hippocampus rather than EC, as the leading 2025 demonstration of causally tested phase-locked memory modulation in humans.

**Table 3:** Quantitative comparison of stimulation modalities

Modality	Target	Reference	Cohort N	Effect size (95% CI)	Behavioral endpoint	Evidence tier
MIMO-driven hippocampal stim.	CA1 (CA3→CA1 codes)	Hampson 2018^4^^,^^5^	*n* = 8 (epilepsy/TBI)	DMS + 37%; DR + 35%; 95% CI not reported	DMS; DR	III
Theta-burst EC microstim.	angular bundle (EC white matter)	Titiz 2017^57^; Suthana 2012^58^	*n* = 7	encoding specificity +20%	spatial nav.; face recognition	III
Theta-phase-locked DBS	hippocampus → LTC	Kragel 2025^31^	*n* = 14	connectivity z + 0.4 (*p* < 0.01)	resting theta-phase synchrony	III
Fornix DBS (130 Hz)	Fornix-BNST junction	Lozano 2016^32^; Ríos 2022^33^	*n* = 42 (mild AD)	primary endpoint negative; ≥ 65 years subgroup positive trend	ADAS-Cog 13	II
tTIS	deep hippocampal	Violante 2023^28^	*n* = 20	≈12% increased recall odds	associative memory	III
Closed-loop TUS	hippocampus (phase-locked)	Yang 2020^115^	proof-of-concept	phase-tracking demonstrated; behavior pending	not reported	IV
tACS	cortical entrainment	multiple^116^^,^^117^^,^^118^	*n* = 10–40	mixed; modest	various memory tasks	III

Evidence tiers: I, multi-center RCT, *N* ≥ 100; II, single-center RCT; III, open-label/case-series, *N* < 50; IV, single case or technical demonstration. Abbreviations: AD, Alzheimer’s disease; ADAS-Cog, Alzheimer’s Disease Assessment Scale-Cognitive; BNST, bed nucleus of the stria terminalis; DMS, delayed match-to-sample; DR, delayed recognition; EC, entorhinal cortex; tACS, transcranial alternating current stimulation; TBI, traumatic brain injury; tTIS, transcranial temporal interference stimulation; TUS, transcranial ultrasound stimulation.

Non-invasive prosthetics include transcranial magnetic stimulation (TMS), transcranial electrical stimulation (tES), and tTIS. tTIS allows selective neuromodulation of deep brain structures: Beanato et al. applied tTIS between hippocampus and EC in continuous or intermittent theta-burst patterns (Figure 4B), achieving improved spatial-navigation performance.^119^ Recent rhythmic sensory and electromagnetic stimulation studies link theta phase-dependent plasticity with episodic memory formation (Figure 4C); theta/alpha transcranial alternating current stimulation (tACS) of the hippocampus/EC enhances early consolidation (Figure 4D).^120^^,^^121^

Focused-ultrasound neuromodulation represents a frontier. Yang et al. developed a closed-loop transcranial ultrasound (CLTUS) system achieving theta-phase prediction via online CA1 LFP monitoring and 500 kHz pulses.^115^ The original study demonstrated real-time theta-phase prediction and acute neural modulation in animals; direct behavioral validation of phase-dependent memory enhancement (including sleep-deprivation effects) remains an open direction. Jo et al. developed a CMUT-based chronic system that solves stimulation-artifact problems, enabling artifact-free recording during stimulation.^122^ Closed-loop acoustic stimulation during sleep uses 50 ms pink-noise pulses at slow-oscillation UP peaks (0.5–1 Hz) to enhance declarative memory^123^; minute timing differences can produce enhancement or suppression of hippocampal ripples,^124^ highlighting the importance of precise phase detection and individualization.

Invasive memory prosthetics implant microelectrode arrays for high-precision detection and stimulation. High-channel-density arrays integrating detection and stimulation form the neuromorphic hardware (Figure 4E).^125^ Miniscope and two-photon microscopy enable simultaneous observation of hundreds-to-thousands of neurons; precise electrical or optogenetic stimulation (Figure 4F) enhances spatial navigation in rodents and primates.^126^^,^^127^ Identification of memory-specific neural features via microelectrodes enables visual-memory improvement^4^; circuit-level mechanisms such as inhibitory plasticity that support replay generalization provide a substrate that electrical, chemical, or optogenetic stimulation may engage to shape memory schemas (Figure 4G).^128^ Cohort sizes, effect sizes, behavioral endpoints, and evidence tiers are compared quantitatively in Table 3.

Infrared neural stimulation (INS) provides high spatiotemporal resolution without genetic modification. Chernov & Roe established its physical basis^129^; Horváth et al. developed integrated multimodal photonic probes.^130^ Zhang et al. showed that 62-day 1070 nm, 10 Hz photomodulation improves working memory in AD mice, with mechanisms involving enhanced theta-gamma PAC.^131^ INS has been demonstrated in non-human primate cortex with single-vessel resolution and sub-millisecond precision^129^^,^^130^; its application to human memory modulation has not been established, and current evidence does not support generalizing efficacy from sensory/motor cortex to hippocampal memory circuits in humans. Magnetogenetics combines deep magnetic-field penetration with cell-type specificity^132^: Lee et al. developed m-Torquer magnetic nanodiscs with Piezo1 for remote control^133^; Choi et al. achieved cell-type targeting compatible with 7T MRI^132^; applications include social-memory modulation and feeding control.^134^ Upconversion-nanoparticle (UCNP) optogenetics addresses the limited penetration of conventional optogenetics: Chen et al. showed that UCNPs convert 980 nm NIR to blue light, transcranially activating photosensitive proteins,^135^ enabling precise fear-memory extraction, hippocampal theta entrainment, and seizure suppression.^136^

Closed-loop regulation creates an “observer-observed” coupling: stimulation changes neural states and may perturb biomarkers that serve as feedback. SWR false-positive accumulation, theta sensitivity to stimulation artifacts, and TGC adaptive decay under sustained stimulation reflect this contradiction. Hybrid architectures separate fast/slow timescales: millisecond phase-locking for instantaneous intervention, hourly behavioral optimization, and day-to-week model updates tracking plasticity—a multi-timescale decoupling that may break the stability/adaptability trade-off.

Design principles for closed-loop control. Six rules follow: (1) reserve fixed-frequency open-loop for invariant biomarkers; use phase-locked/event-triggered closed-loop for biomarkers varying on theta-cycle (∼125 ms) or task-relevant timescales; (2) match paradigm to dynamics—MPC when a tractable forward model exists; RL when dynamics are non-stationary and on-chip plasticity supports per-trial updates; (3) avoid pseudo-closed-loop architectures where signals do not gate stimulation in real time; (4) hard-code biphasic charge balance and per-pulse limits (typically ≤ 100–150 μC cm^−2^ for IrOx, Pt-Ir, PEDOT composites; residual DC < 1% of pulse amplitude) at the stimulator front end; (5) treat patient-specific MIMO/Volterra fits as having finite useful lifetime—plan recalibration every 2–4 weeks with ≥ 100–500 successful encoding-retrieval pairs per session; and (6) integrate stimulation-artefact blanking with sub-millisecond recovery, since same-electrode record-stimulate (as in conformal MEAs^64^^,^^65^^,^^66^^,^^67^^,^^68^) requires a clean post-pulse window <5 ms before the next theta-phase decision.

Implications for materials SPP. The two paradigms (Table 2) place complementary, partially conflicting demands. Model-predictive MIMO control requires repeated matrix operations within a theta cycle (∼125 ms), favoring non-volatile memristors with retention > 10^5^ s and endurance > 10^9^ cycles. RL controllers require on-chip plasticity with linear/symmetric LTP/LTD, driving advances in HfO_2_- and TaO_x_-based filamentary memristors and SrTiO_3_ oxygen-vacancy systems. DBS/network modulation abstracts away from spike-level content and prioritizes chronic macroelectrode stability over computational density. Theta-phase-locked closed-loop protocols^31^ and fornix DBS^32^^,^^33^ demand low-impedance, high-CIC electrodes delivering charge-balanced biphasic pulses at 30–100 μA without exceeding 100–150 μC cm^−2^—anchoring coating choice to IrOx, Pt-Ir, and PEDOT composites.

## Computational models and decoding algorithms: from signal processing to adaptive encoding

Computational models and signal processing algorithms constitute the algorithmic backbone of memory prosthetic systems, decoding memory states and generating stimulation patterns. The evolution from classical filters to deep learning has improved decoding accuracy and real-time performance, establishing the foundations for closed-loop neural prosthetics.

### Decoding algorithms: From classical filters to deep learning

**Figure 5:**
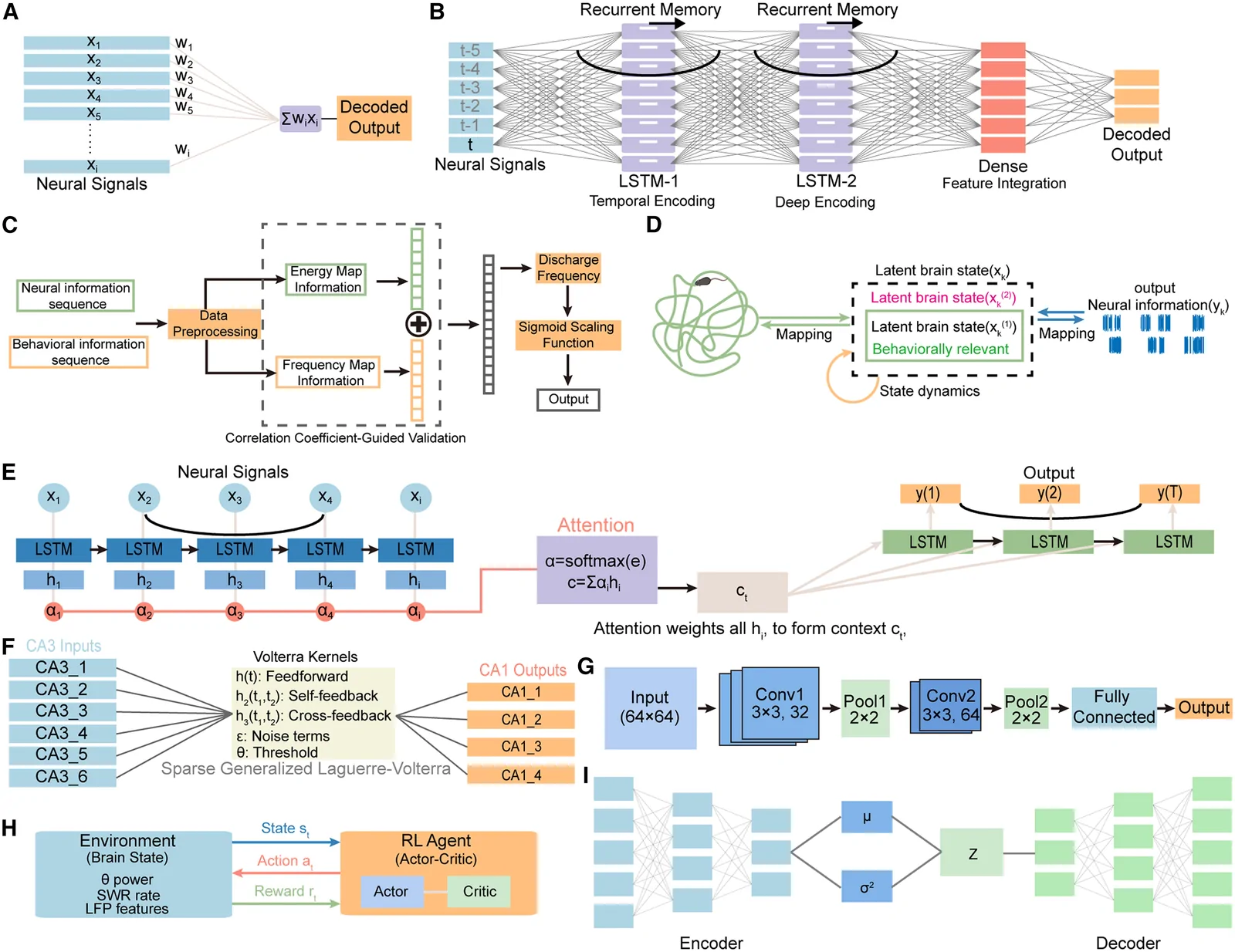
Neural network architectures for memory encoding and decoding (A) Feedforward/state-space network (schematic basis for Kalman-filter decoding): input x maps to output ŷ through hidden layer h. (B) Recurrent network (schematic basis for LSTM and other RNN-based decoders): temporal recursive connections enable sequence prediction. (C) Decoding and feedback electrical-stimulation output. (D) DPAD framework. (E) Encoder-decoder with attention: encoder maps x to hidden states h; attention computes weights α and context c; decoder generates y. (F) MIMO Volterra model describing the nonlinear CA3→CA1 transformation. (G) CNN with Conv/Pool layers for spatial pattern recognition. (H) RL: Actor selects a from state s; Critic evaluates state value; updates use policy gradient and TD error; reward r encodes memory performance. (I) VAE: encoder maps x to latent (μ, σ^2^); z is sampled via reparameterization; decoder reconstructs.

Neural decoding has evolved from classical signal processing to deep learning. Stationary wavelet transform achieves real-time action-potential detection via translation invariance^137^; wavelet filtering improves spike SNR and clustering.^138^ The Kalman filter (Figure 5A) anchors classical decoding: Wu et al. established a linear dynamic model for real-time motor-intention mapping^139^; unscented Kalman filters handle nonlinear tuning^140^; ReFIT-KF online learning adapts to signal drift.^141^ Particle filters handle nonlinear, non-Gaussian characteristics, with Liu et al. achieving 38.5-min warning in epilepsy prediction.^142^ Machine learning and deep learning extend nonlinear decoding^143^^,^^144^: LSTM networks (Figure 5B) decode motor information from calcium imaging with high speed^145^ (Figure 5C); DPAD (Figure 5D) performs nonlinear neural dimensionality reduction while preserving behavioral information^146^; attention-based architectures enable interpretable decoding (Figure 5E)^147^; RNNs in hippocampal position decoding achieve errors (7.7 cm) better than Bayesian (10.4 cm).^148^

### MIMO models for hippocampal memory encoding

MIMO models represent one of the most successful translations of hippocampal neurophysiology into prosthetics (Figure 5F).^4^^,^^30^^,^^126^ The MIMO program originated with Song, Berger and colleagues, who established the Volterra-kernel approach in a series of foundational studies: a MIMO nonlinear model of CA3–CA1 dynamics^149^; point-process spike-train prediction through a generalized Laguerre-Volterra framework^150^; and sparsification reducing free parameters by an order of magnitude while preserving accuracy.^151^ The resulting sparse generalized Laguerre-Volterra framework precisely simulates CA3→CA1 information transformation, with feedforward/feedback Volterra kernels, endogenous noise, sub-threshold dynamics, and spike-threshold functions.^152^ Validation in 8 human patients with epilepsy via cross-validation and Kolmogorov-Smirnov tests confirmed accurate CA1-spike prediction from CA3 activity.^153^ The key innovation is extracting patient-specific stimulation patterns from successful encoding trials; MIMO-derived patterns significantly outperform random equal-strength/frequency stimulation.

For optimization, Song et al. introduced sparse regularization via group lasso and local coordinate descent^154^; Roy et al. used CNNs for fast real-time decoding (Figure 5G).^155^ She et al. developed the double-layer multi-resolution classification framework—the algorithmic core of the memory decoding model (MDM)—which solves the underdetermined problem at small clinical sample sizes by combining B-spline expansion, L1-regularized logistic classifiers, and stacked ensembles across temporal resolutions, enabling robust single-trial decoding of spatiotemporal spike patterns.^156^ Building on this, Roeder et al. extended MDM for specific information recall, creating engram-based stimulation codes.^5^ A critical translational bottleneck is model-estimation time: full multi-channel MIMO fitting requires hundreds of hours on a single workstation. She et al. addressed this with parallel MISO sub-model estimation across HPC nodes, reducing total time to within 72 h—the clinical constraint of human-trial protocols—demonstrating that on-demand patient-specific MIMO models are operationally feasible.^157^ First-in-human trials of MIMO stimulation in patients with TBI showed improvements in delayed-recall and delayed-recognition under stimulation.^36^^,^^158^ Most recently, She et al. applied the MDM framework to 24 patients with epilepsy in a visual DMS task, decoding visual-memory categories—the “what” dimension of episodic memory—from hippocampal CA3/CA1 spiking with significantly above-chance accuracy.^159^ Critically, individual neurons used a distributed temporal code (not a rate code) for object categories, and CA3/CA1 carried largely redundant category information due to strong feedforward connectivity—findings that directly inform stimulus-pattern design for next-generation category-selective MIMO prosthetics.

Context-dependent nonlinear interactions are captured through task-specific firing patterns.^160^ Deadwyler et al. demonstrated closed-loop functional-ensemble stimulation in non-human primates by extracting features from correctly completed trials.^126^ Neural firing patterns may differ markedly across cognitive contexts,^160^ requiring dynamic adaptation via continuous MIMO coefficient updating or RL-based stimulation-parameter adjustment.^161^^,^^162^^,^^163^

### Network-level and mechanism-based models

Network-level modeling integrates place cells, grid cells, and MTL architecture. Giocomo et al. showed that excitatory/inhibitory synaptic plasticity forms place- and grid-cell tuning on behavioral timescales^164^; Cluster and memory-based models propose that place/grid-cell organization can emerge from domain-general learning or memory processes rather than dedicated navigation circuits,^165^^,^^166^ suggesting that the MTL may employ general computational algorithms transferable across domains. We note that place- and grid-cell coding is most prominent in rodent spatial cognition and is of reduced direct significance for human declarative memory; these mechanisms are therefore included as mechanistic constraints rather than as direct decoding targets for human prosthetics. Computational models of SWR generation integrate refractory periods, stochastic initiation, exponential CA3 accumulation, and PV+ basket-cell mutual inhibition for ripple frequency^167^^,^^168^; optogenetic silencing confirms PV + cells' critical role.^169^ This understanding provides intervention targets: enhancing PV + activity may extend ripples (better consolidation),^170^ while disrupting them eliminates ripples.^167^ Direct clinical translation requires caution because non-specific PV + manipulation may interfere with gamma oscillations.^171^

### Adaptive control: Model-predictive and reinforcement learning

Model-predictive control (MPC) enables precise stimulation by predicting future states and optimizing control sequences.^172^ Kirsch et al. developed gradient-projection MPC for functional electrical stimulation with steady-state RMS error <2°.^173^ Hussain et al.'s AxonML combines neural networks with MPC, achieving 90,000-fold acceleration of peripheral-nerve stimulation modeling and making real-time nonlinear MPC feasible.^174^

Reinforcement learning (RL) provides a powerful basis for adaptive closed-loop neuromodulation (Figure 5H). Lu et al. and Cho et al. applied Actor-Critic algorithms to DBS, achieving 63.3–67% energy reduction while maintaining therapeutic effect.^175^^,^^176^ Cho et al. compared SAC, TD3, PPO, and A2C, finding TD3 performed best.^176^ Ravivarapu et al. (2025) proposed SEA-DBS, integrating a predictive reward model to reduce real-time-feedback reliance and Gumbel-Softmax exploration for stable updates—addressing sample complexity and hardware deployability for RL-based neurostimulation.^177^ State space comprises LFP features (power spectral density, beta-band power)^178^^,^^179^; actions include stimulation amplitude, frequency, pulse width^176^; reward functions balance symptom relief and energy penalties. Transferred to memory prosthetics: state features include theta power, TGC, and SWR rate^83^^,^^161^; actions correspond to stimulation parameters; reward is built from encoding-success rate, retrieval accuracy, and safety constraints (charge-density limits).^162^^,^^163^ Variational autoencoder (VAE) disentangled representation learning improves interpretability (Figure 5I).^180^ At the single-neuron level, skew-t mixture-model spike sorting achieves *R*^2^ = 0.98.^181^

### Paradigm mapping and design implications

MIMO success demonstrates the effectiveness of biomimetic encoding, but the “black-box” nature of deep models conflicts with neurophysiological transparency. This raises the paradigm choice: replicate hippocampal dynamics via spiking neural networks (interpretability, biocompatibility) or leverage Transformers for supra-biological capabilities. Transfer learning offers a middle path—pre-train on large-scale neural data, fine-tune for individuals—balancing generalization with personalization.

Read against the Section-1 paradigm distinction (Table 2), the algorithms in this section sit within the MIMO/information-processing branch: Volterra-kernel MIMO encoders, deep recurrent networks, attention-based sequence models, and RL controllers all reconstruct, decode, or modulate specific information flow at the spike or spike-train level. The DBS/network-modulation branch operates on macroscopic state variables (theta power, theta-trough phase, TGC, SWR rate), so its “decoders” are compact thresholding or state-estimation modules rather than large parametric networks. The division is one of intervention layer, not methodological rigor, and maps onto distinct hardware constraints: high-channel-count microelectrode arrays with on-chip neuromorphic acceleration for MIMO-class systems vs. compact phase-locked loops and chronically stable macroelectrode leads for DBS-class systems. Hybrid prosthetics that combine a MIMO encoder with a DBS-style outer loop (e.g., theta-trough-gated delivery of MIMO patterns) are a natural design synthesis.

Design rules. (1) Cap Volterra kernel order at three for 16-channel CA3→CA1 encoders—higher orders exceed the ∼10^5^ ceiling of 2D TiO_2-x_/Al_2_O_3_ crossbars at sub-mW power^149^^,^^150^^,^^151^; (2) match decoder parameter budget to hardware density (∼10^5^ for 2D crossbars; 10^6^–10^7^ only for 3D-stacked architectures with anneal < ∼400 °C); (3) prefer interpretable sparsity-regularized decoders (Volterra-L1, Bayesian state-space, Kalman) for clinical/regulatory contexts; reserve deep architectures for accuracy-bound, data-rich applications; (4) plan ≥100–500 successful encoding-retrieval pairs per patient for MIMO fitting; fall back to transfer learning with population priors when data are insufficient; (5) apply L1 sparsification to MIMO models^151^; (6) partition decoders into static blocks (high-density interfacial memristors) and online-update blocks (filamentary HfO_2_/TaO_x_ devices).

Materials implications. Fitting a 16-channel CA3→CA1 MIMO with Volterra kernels up to order three requires ∼10^5^ weights^149^^,^^150^^,^^151^; today’s 2D TiO_2-x_/Al_2_O_3_ memristive crossbars at the 100 nm node reach this density at sub-mW static power, two orders below CMOS SRAM-equivalent designs. Deep recurrent and Transformer-class decoders raise the budget to 10^6^–10^7^, demanding 3D-stacked memristive layers (anneal < ∼400 °C to preserve TSV integrity) or aggressive sparsification. RL controllers add a third constraint: on-chip local weight updates favor filamentary devices with linear/symmetric LTP/LTD over filament-free interfacial switches. The decoder-family choice flows back into specifications on synaptic-device chemistry, layer count, and CMOS-BEOL compatibility—addressed quantitatively in Section materials design and hardware implementation: from Memristive synaptic devices to neuromorphic platforms.

## Materials design and hardware implementation: from memristive synaptic devices to neuromorphic platforms

This section addresses the materials-design layer. The treatment is organized around the structure-property-processing (SPP) trilemma and covers chronic electrode arrays and conformal substrates, together with the electrochemical and biocompatibility properties they enable; synaptic devices for neuromorphic computation; emerging optical, magnetic, and ultrasonic modalities; and validated neuromorphic demonstrations. The organizing claim is that no single materials family satisfies all three engineering specifications (low-noise chronic recording, safe high-density stimulation, on-chip personalized computation); clinically deployable systems must combine families site by site and layer by layer. Representative anchors from Materials & Design and adjacent venues include the 2D perovskite memristive substrates of Xiao et al.^182^ and the conformal hippocampal MEAs of Xu, Wang, Elyahoodayan and colleagues,^64^^,^^65^^,^^66^^,^^67^^,^^68^ whose SPP chains are made explicit later in discussion.

### Electrode materials for chronic neural interfaces

Chronic interfaces face a triple constraint: low impedance for high-SNR recording, high CIC for safe stimulation, and minimal foreign-body response over months to years. Smooth-Pt CIC (0.05–0.15 mC cm^−2^) is insufficient for the 30–100 μA amplitudes of hippocampal phase-locked protocols.^31^ Three platforms address the gap. (1) Pt-Ir alloy and sputtered IrOx coatings raise CIC into 1–4 mC cm^−2^; sputtering pressure, oxygen partial pressure, and post-anneal temperature control porosity and hydration. (2) PEDOT:PSS and PEDOT/CNT composites, electropolymerized at 5–10 mM, reduce impedance by 1–2 orders of magnitude at 1 kHz with CIC 2–15 mC cm^−2^; chronic delamination at polymer-metal interfaces is addressed by covalent silane priming, with >12-month *in vivo* stability demonstrated.^183^ (3) Carbon-based electrodes (laser-scribed C, CNT-fibre, graphene) provide a 3D porous matrix with brain-matched modulus (≈0.5–2 MPa vs. ≈ 200 GPa for metals), reducing glial encapsulation. No single chemistry satisfies all three constraints; cMEAs combine them through site-by-site selection. Xu, Wang, and Elyahoodayan’s cMEA work^64^^,^^65^^,^^66^^,^^67^^,^^68^ integrates parylene-C substrates with IrOx/Pt-Ir coatings for 16–64 channel coverage matched to MIMO requirements. At the fiber-processing scale, Zhou et al. quantified how floating-catalyst CVD parameters govern CNT length and bundle alignment, with the resulting tensile-strength/conductivity envelope setting a fabrication-side bound on chronic CNT microelectrode fiber diameter.^184^

### Flexible and conformal bioelectronic interfaces

Modulus mismatch between rigid silicon probes and brain tissue is the dominant driver of chronic-recording failure: > 3 orders of magnitude generates micromotion shear that triggers astrocyte/microglia recruitment within 2–4 weeks, degrading recording quality 30–60% within 6 months.^185^ Three strategies have emerged: (1) thin-film polyimide and parylene-C (5–20 μm) reduce bending stiffness >3 orders of magnitude vs. silicon; transfer printing onto biodegradable silk fibroin provides temporary insertion-time stiffness that dissolves post-implant^186^; (2) mesh-electronics architectures with tissue-matched feature pitches (≈100 μm) achieve seamless integration with stable single-unit yield over 8 months in rodent hippocampus^187^; (3) liquid-metal/elastomer hybrids (Galinstan-in-PDMS) and MXene-coated stretchable substrates have brain-matched moduli (0.1–1 MPa) but face saline-stability challenges. Ye et al. reported in Materials & Design a self-limited ns-UV-laser-sintering route in which spray-coated Ga-based liquid-metal sub-micron particles are sintered to μm-thick films, reducing sheet resistance >7 orders of magnitude to ∼2 Ω sq^−1^ without damaging soft substrates^188^; this provides an explicit processing-structure-property chain transferable to liquid-metal microelectrodes on parylene-C/polyimide carriers, with *in vivo* biocompatibility remaining to be validated. The cMEA work of Xu, Wang, Elyahoodayan and colleagues^64^^,^^65^^,^^66^^,^^67^^,^^68^ demonstrates feasible 16–30 channel same-electrode record-stimulate operation with parylene-encapsulated Pt-IrOx contacts.

### Memristive materials for synaptic emulation

**Figure 6:**
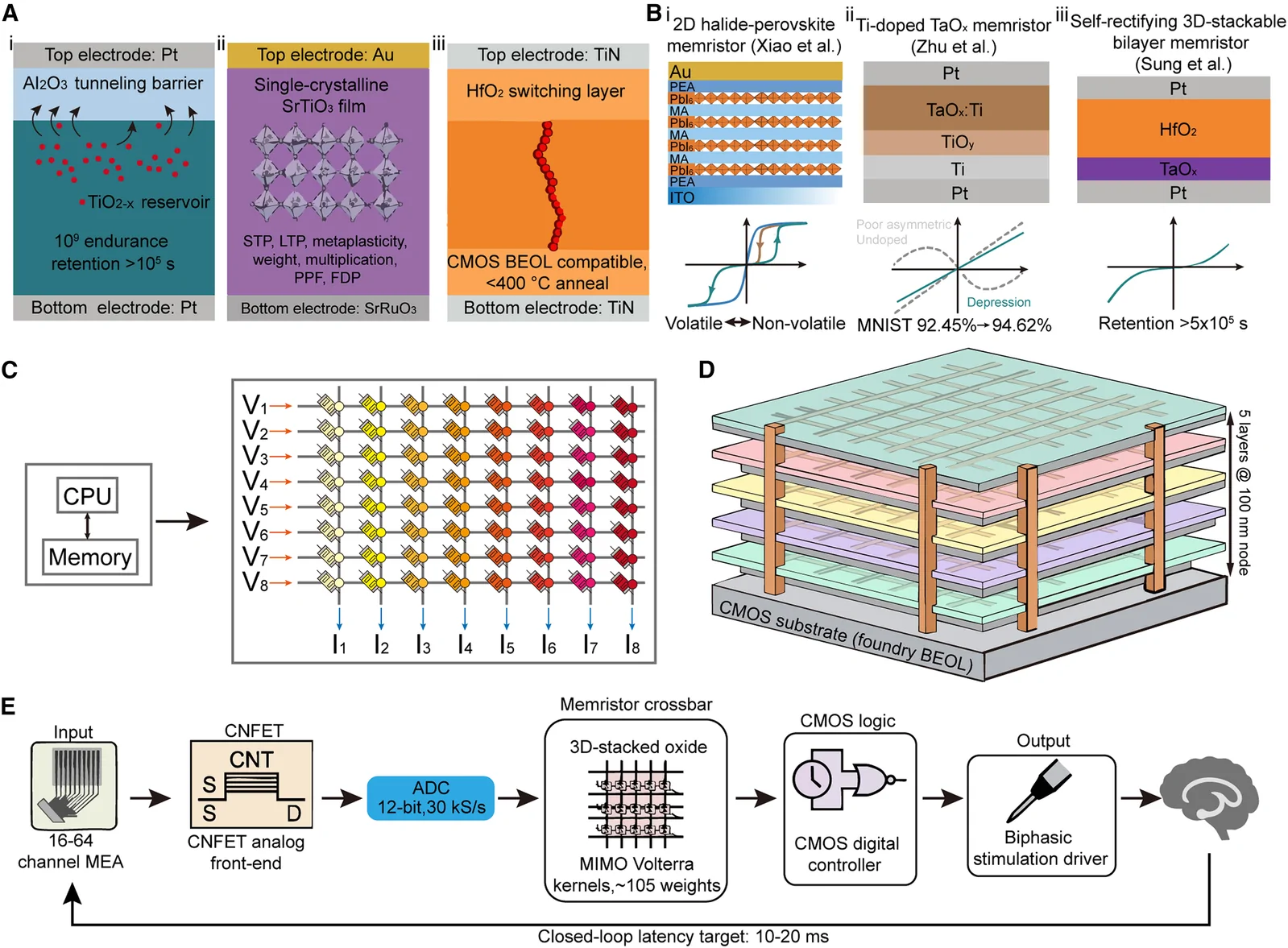
Memristive devices and their integration into neuromorphic platforms for hippocampal memory prosthetics (A) Three dominant oxide-memristor families: (i) Al_2_O_3_/TiO_2-x_ bilayer (oxygen-vacancy migration; 10^9^-cycle endurance; retention > 10^5^ s at 85 °C)^189^; (ii) SrTiO_3_ perovskite (six co-existing synaptic behaviors in one device)^190^^,^^191^; (iii) HfO_2_ filamentary (CMOS BEOL compatibility; < 400 °C anneal).^192^ (B) Three Materials & Design anchor devices: (i) 2D (PEA)_2_MA_3_Pb_4_I_13_ halide-perovskite memristors switching between volatile/non-volatile by compliance-current alone, supporting LIF neuronal excitation and long-term plasticity within one device^182^; (ii) 8 at.% Ti-doped TaO_x_ memristors widening the switching window an order of magnitude with near-linear LTP/LTD curves^193^; (iii) self-rectifying Pt/TaO_x_/HfO_2_/Pt selectorless bilayer with retention > 5 × 10^5^ s, rectification ratio ∼2.3 × 10^3^, cycle-to-cycle variability < 0.022.^194^ (C) Crossbar in-memory computing collapses MVM into a single Ohm-plus-Kirchhoff step.^189^ (D) 3D vertical stacking of 5 memristive layers at 100 nm increases synaptic density 5× without footprint penalty^195^^,^^196^; the principal processing constraint is BEOL anneal < ∼400 °C for TSV integrity. (E) Heterogeneously integrated neuromorphic memory-prosthetic platform combining parylene-substrate cMEA (PEDOT:PSS/IrOx/Pt-Ir contacts),^64^^,^^65^^,^^66^^,^^67^^,^^68^ a CNT-FET analog front-end with stimulation-artefact blanking, a 3D-stacked oxide-memristor crossbar storing patient-specific MIMO Volterra kernels (∼10^5^ weights), CMOS digital control, and a charge-balanced biphasic driver bounded by the 150 μC cm^−2^ Shannon-Pudenz limit, operating within a 10–20 ms closed-loop latency budget.

Memristive devices reproduce analog, non-volatile, history-dependent conductance modulation, making them the most actively developed substrate for prosthetic neuromorphic computation. Three families dominate (Figure 6A). (1) Al_2_O_3_/TiO_2-x_ bilayer heterostructures achieve resistive switching through oxygen-vacancy migration between an Al_2_O_3_ tunnel barrier and TiO_2-x_ reservoir; the bilayer decouples set/reset kinetics, yielding > 100-level conductance, 10^9^-cycle endurance, retention > 10^5^ s at 85°C^189^. (2) SrTiO_3_ perovskite memristors exploit oxygen-vacancy switching in single-crystalline films; the perovskite ABO_3_ TiO_6_-octahedron lattice supports six co-existing synaptic behaviors (short/long-term plasticity, metaplasticity, weight multiplication, paired-pulse facilitation, frequency-dependent plasticity) within one device.^190^^,^^191^ (3) HfO_2_-based memristors offer CMOS-process compatibility for ASIC integration with reliable low-energy switching.^192^ The most consequential SPP linkage for prosthetics is between switching uniformity (determined by filament-formation stochasticity, controlled by deposition thickness, anneal temperature, electrode chemistry) and MIMO fidelity: cycle-to-cycle variability > ∼5% degrades multi-channel decoding accuracy, setting fabrication specifications stricter than for general AI accelerators (ALD thickness control ± 0.5 Å, anneal uniformity ± 5 °C, electrode stoichiometry ± 1 at.%) only recently achievable at foundry grade. Xiao et al. recently demonstrated in Materials & Design that 2D (PEA)_2_MA_3_Pb_4_I_13_ organic-inorganic hybrid perovskite memristors switch between volatile and non-volatile resistive states by compliance-current tuning alone, supporting leaky-integrate-and-fire excitation and long-term plasticity within one device whose SPP is governed by 2D-perovskite thickness, halide-ion migration, and electrode chemistry.^182^ Zhu et al. demonstrated in Materials & Design that 8 at.% Ti co-sputtered into TaO_x_ widens the resistive-switching window by an order of magnitude (> 10^3^ vs. 10^2^) and yields near-linear LTP/LTD (nonlinearity 1.12/1.01, symmetricity 43.56), lifting MNIST accuracy from 92.45% to 94.62%,^193^ directly tracing the dopant-concentration (processing) → oxygen-vacancy density (structure) → LTP/LTD linearity (property) chain (Figure 6B).

### Neuromorphic architectures and system integration

Clinically deployable systems must satisfy three constraints: power < 100 mW (Pennes-equation thermal limit), latency <20 ms for theta-phase locking, and footprint < 1 cm^2^ for sub-galeal enclosure. Crossbar in-memory computing addresses all three: MVM collapses from O(N^2^) operations to a single Ohm/Kirchhoff step, eliminating data-movement energy (Figure 6C). 3D vertical stacking (5 layers at 100 nm node) increases synaptic density 5× without footprint penalty^195^^,^^196^ (Figure 6D) but introduces the TSV thermal-budget challenge driving low-temperature (< 400 °C) anneal HfO_2_ films. Heterogeneous integration with CNT field-effect transistors and CMOS logic^197^ provides readout, threshold-detection, and event-driven control (Figure 6E). The principal architecture-materials challenge is on-chip plasticity for personalization: patient-specific MIMO models (including Song/Berger Volterra formulations^149^^,^^150^^,^^151^) evolve over weeks and require local weight updates without external compute, achievable today only through memristors with linear/symmetric LTP/LTD characteristics. Sung et al. demonstrated in Materials & Design that a 3D-stackable Pt/TaO_x_/HfO_2_/Pt selectorless memristor can be engineered into a self-rectifying multilevel cell by matching the dielectric capacitances of the two oxide layers, concentrating bias across HfO_2_ while TaO_x_ acts as a built-in rectifier; the resulting devices show retention > 5 × 10^5^ s, on/off and rectification ratios ∼1.35 × 10^3^ and 2.3 × 10^3^, multilevel programmability, and cycle-to-cycle variability < 0.022, suppressing sneak-path current that has historically capped 3D stack size.^194^ Aligning with the journal’s remit, Xiao et al.'s 2D halide-perovskite memristors^182^ further illustrate how a single solution-processed stack can satisfy two system constraints simultaneously: compliance-current tuning switches between volatile and non-volatile regimes, collapsing the neuron/synapse functional partition that has historically driven heterogeneous integration.

### Materials-enabled optical, ultrasonic, and magnetic interfaces

Three emerging modalities open new materials-design problems. UCNP-optogenetics enables NIR-excited mm-deep activation of channelrhodopsin-expressing neurons; the UCNP itself (typically NaYF_4_:Yb^3+^,Er^3+^/Tm^3+^) must be processed into ≈20 nm crystallites with controlled surface chemistry to combine upconversion efficiency > 0.5%, aqueous colloidal stability, and biocompatibility, the last remaining the principal open question for chronic human use. Magnetothermal and m-Torquer iron-oxide nanoparticles convert remote magnetic fields into local heating or torque to activate TRPV1 or mechanosensitive neurons; the materials problem reduces to controlling size distribution (15–25 nm core) and surface coating to achieve the specific absorption rate needed for sub-degree heating without runaway injury. Focused-ultrasound CMUTs integrate piezoelectric MEMS lithography to deliver phase-locked ultrasound to the hippocampus; recent system demonstrations^115^ establish closed-loop phase tracking, with behavioral memory outcomes remaining to be validated in human studies. These modalities share the SPP logic described in the preceding electrode- and memristor-focused subsections but operate in regimes (nanocrystalline lanthanide hosts, ferrite nanoparticles, piezoelectric MEMS) demanding entirely different fabrication infrastructures.

### Existing neuromorphic demonstrations and validated material systems

Three established demonstrations validate the materials choices above. Neuromorphic circuits provide energy-efficient hardware for memory prosthetics,^198^^,^^199^ with performance governed by SPP relationships of constituent memristive materials. These circuits use ANN with memristor synapses exhibiting graded LTP/LTD^200^^,^^201^; switching arises from ion migration and filament formation in metal-oxide thin films, with composition, crystallinity, and interface engineering determining synaptic weight precision, endurance, and retention. Crossbar architectures support analog/in-memory computation at high density. At the device level, SrTiO_3_ memristors implement six synaptic functions within one device—short/long-term memory, short-term plasticity, metaplasticity, and weight multiplication; the perovskite oxygen-vacancy resistive switching with analog tunability achieved 67% power reduction in RL Atari Pong tasks vs. open-loop.^190^^,^^191^

Large-scale neuromorphic systems advance through innovations in processing and 3D architectures.^202^ Kataeva et al. demonstrated deep ANN chips with 2.4 million Al_2_O_3_/TiO_2-x_ memristors—the bilayer providing complementary switching (Al_2_O_3_ tunneling barrier, TiO_2-x_ vacancy reservoir) with ∼1000× complexity over previous designs.^189^ 3D integration achieved 5-layer 100 nm memristor structures via vertical stacking,^195^^,^^196^ an advance in nanoscale processing for high-density computing. These can be integrated with CNT FETs and CMOS logic^197^ for heterogeneous hybrid architectures. Memristor-based reservoir computing uses short-term dynamics for spatiotemporal processing without backpropagation through time.^203^^,^^204^ SNNs directly implement STDP in hardware^205^^,^^206^ for event-driven, energy-efficient computation,^198^^,^^199^ with temporal dynamics matching biological systems.^207^^,^^208^

Neuromorphic hardware offers multiple advantages: (1) ultra-low power from analog switching in metal-oxide/perovskite memristors; (2) real-time processing from fast ionic kinetics in nanoscale films; (3) adaptive learning from voltage-dependent conductance modulation; (4) high scalability from nanofabrication; and (5) co-localized computation/storage from non-volatile resistive states, eliminating von Neumann bottlenecks. Whereas implantable systems are limited by battery life and thermal dissipation, neuromorphic approaches promise 2–3 orders of magnitude power reduction, enabling chronic autonomous operation.

## Conclusions and future directions

Memory prosthetic technology stands at a critical juncture in transition from laboratory validation to clinical deployment. The year 2025 has witnessed pivotal advances: the first causal demonstration that closed-loop theta-synchronized stimulation persistently enhances human hippocampal network connectivity,^31^ the discovery of coordinated excitatory-inhibitory entorhinal inputs that stabilize memory maps,^13^ and breakthroughs in engram-targeted cellular reprogramming demonstrating potential to rescue age-related cognitive decline.^42^ With first-in-human trials demonstrating 35–37% memory improvement in small cohorts,^4^^,^^5^ the field has advanced from conceptual exploration to early clinical translation; broader generalization requires larger trials. This milestone builds on half a century of neuroscience—from Tulving’s episodic-memory framework^6^ through place and grid cells^8^^,^^75^ to theta oscillations and SWRs^11^^,^^12^—now translated into theta phase-locked algorithms, SWR-triggered protocols, and patient-specific MIMO encoders. Equally critical, advances in memristive materials, nanoscale electrode fabrication, and neuromorphic architectures provide the hardware substrate for chronic implantation.

Cross-scale principles emerge with attention to study scope. Anatomically, in encoding-phase modulation of the perforant pathway, theta-burst stimulation of EC white matter (angular bundle) produced larger memory-specificity gains than gray-matter stimulation under matched currents in selected studies^57^^,^^58^; fornix-BNST stimulation has been associated with greater clinical benefit than isolated fornix stimulation in AD subgroup analyses^33^; and connectome-based individualized targeting outperforms stereotyped coordinates.^33^ These observations are paradigm- and task-specific rather than universal. Temporally, theta-trough-locked stimulation before/during encoding has been associated with stronger encoding enhancement than retrieval-phase or random timing in selected paradigms^61^; engineering targets in 10–20 ms are pursued for theta-phase locking and SWR-triggered intervention,^62^^,^^63^^,^^102^ with tolerable latency depending on theta cycle and required phase precision. Stimulation patterns: patient-specific MIMO encoding patterns outperform fixed-frequency in first-in-human cohorts^4^^,^^30^^,^^126^; theta-burst patterns outperform continuous high-frequency in encoding-phase protocols^57^^,^^58^; microelectrode μA-level current is preferable to macroelectrode mA-level stimulation.^57^ Biomarker monitoring: multimodal monitoring (theta power, theta-gamma PAC, SWR rate, HFA) is preferred over single metrics; content decoding enables selective manipulation.^106^^,^^107^

Choice between invasive and non-invasive approaches is a clinical decision. Invasive DBS provides single-neuron resolution decoding, precise closed-loop control, direct hippocampal/EC access, and real-time modulation, but with surgical risks and high cost^209^—appropriate for severe deficits (advanced AD, severe TBI, treatment-resistant epilepsy with memory disorders). Non-invasive methods such as TI (the principle underlying tTIS) provide ∼12% increased recall odds and target deep hippocampal structures with good tolerability and reversibility but lower spatial precision and smaller effect sizes.^28^ Recent advances are substantial: hippocampal TI modulates task-related resting-state connectivity^117^; TI suppresses epileptic biomarkers in mesiotemporal epilepsy with strong carry-over^210^; comprehensive reviews establish TI as a clinically viable non-invasive deep-brain modality.^116^^,^^118^ Non-invasive methods suit mild-to-moderate impairment, the elderly with surgical contraindications, pediatric cases, and resource-limited settings. The principle is to match architecture complexity and invasiveness to disease severity, prioritizing non-invasive unless functional needs justify surgical risk. Table 4 summarizes the comparison.

**Table 4:** Comparison of invasive and non-invasive memory prosthetic approaches

Parameter	Invasive (DBS, MIMO)	Non-invasive (tTIS, TUS, tACS)
Spatial resolution	high (single-neuron to mm)	low to moderate (cm scale)
Effect size	35–37% memory improvement^4^^,^^5^	12% increased recall odds^28^; variable across modalities
Safety profile	surgical risks (infection, hemorrhage)	minimal (transient sensations)
Reversibility	limited (device removal required)	fully reversible
Cost	high (comparable to DBS systems)	low to moderate
Target population	severe, refractory cases	mild-to-moderate impairment
Closed-loop capability	fully supported	limited (emerging)

DBS, deep brain stimulation; tTIS, transcranial temporal interference stimulation; TUS, transcranial ultrasound stimulation; tACS, transcranial alternating current stimulation.

Unresolved challenges shape the research frontier. Technically: can current 10–35 ms closed-loop latency be reduced below 5 ms? When will wireless multi-site implantable systems be clinically feasible? Can on-chip AI achieve truly real-time content decoding? Theoretically: do current MIMO models capture the full dynamics required? Can spatial-memory models generalize to non-spatial domains? How do adaptive controllers interact with continuous learning? Clinically: which patient subgroups benefit most? Optimal intervention windows (early vs. late dementia)? Combination with pharmacotherapy? Long-term side effects and adaptation? Next-generation systems will integrate multimodal biomarker monitoring (theta/gamma/SWR/HFA), millisecond-precision phase-locking and content-selective stimulation, patient-specific MIMO/MDM, flexible bioelectronic interfaces (graphene, conducting polymers, shape-memory alloys), on-chip neuromorphic processing on memristive materials, bilateral hippocampal-cortical targeting, RL-based adaptive control, and ethical/regulatory frameworks protecting autonomy and privacy.

Several emerging frontiers are poised to reshape the field: (1) Transformer-based decoding may improve temporal resolution and accuracy over current RNNs; (2) digital-twin patient-specific computational replicas enable in-silico parameter optimization; (3) federated learning enables multi-centre model improvement while preserving privacy; (4) explainable AI methods provide transparent rationales for stimulation choices—a prerequisite for regulatory approval; (5) next-generation memristive materials (2D TMDCs, organic-inorganic perovskites, ferroelectric HfO_2_) promise to overcome endurance/variability limits; (6) edge-computing/neuromorphic architectures may achieve sub-5 ms latency at ultra-low power. The challenge has shifted from “can we create memory prosthetics?” to “how do we optimize signal processing, materials platforms, and control architectures for maximum safety, efficacy, and accessibility?” Ethical, regulatory, and translational dimensions, treated as integral to design rather than post-hoc constraints, are addressed in Section ethical, regulatory, and translational considerations.

## Ethical, regulatory, and translational considerations

The trajectory of memory prosthetics is shaped by ethical, regulatory, and translational factors as much as by technical advances. Reading and modifying episodic memory at the circuit level raises questions that signal-processing metrics alone cannot resolve: who decides which memories are enhanced, suppressed, or restored; how informed consent is obtained for technologies whose long-term effects on personal identity remain incompletely characterized; and through which regulatory pathways clinical adoption should proceed. Treating these as integral to design rather than post-hoc constraints is essential for clinical viability.

Therapeutic applications for restoring pathological memory deficits have ethical consensus and clear regulatory pathways for AD, TBI, epilepsy, and stroke. Cognitive-enhancement applications for healthy individuals remain controversial, raising concerns about personal identity (are artificially enhanced memories “truly mine”?), autonomy (AI-driven stimulation may conflict with intentions), social justice (cognitive inequality), and coercion (workplace/educational pressure). Erden and Brey proposed six cognitive-enhancement ethical principles emphasizing non-dependence as a job requirement, genuine well-being, informed consent, justice, autonomy, and social responsibility.^211^^,^^212^ Regulatory agencies (FDA, EU MDR) have approval pathways for therapeutic devices but lack frameworks for enhancement. Researchers must transparently report risks, ensure participant diversity, implement long-term follow-up, and protect data security.

Translationally, the route from first-in-human demonstrations^4^^,^^5^ to broadly accessible therapy requires coordinated progress across multiple fronts. Regulatory pathways for closed-loop adaptive devices remain less mature than for fixed-parameter neurostimulators, with adaptive algorithms posing novel validation questions about certifying devices whose parameters evolve during chronic use. Equitable access is a parallel concern: implantable closed-loop systems with patient-specific MIMO models impose substantial cost and infrastructure requirements that risk concentrating availability in well-resourced centers. Non-invasive alternatives such as TI stimulation^116^^,^^117^^,^^118^^,^^210^ may broaden access at the cost of smaller effect sizes (Table 4). Multi-stakeholder governance—engaging patients, clinicians, regulators, ethicists, and engineers from early design—complements rather than replaces the materials, signal-processing, and control advances of Sections introduction, target selection and temporal control: engineering constraints from neuroanatomy, real-time neural decoding: signal processing pipelines for memory-state detection, behavioral validation of neural biomarkers: closed-loop performance metrics, closed-loop control architectures: from biomarker detection to adaptive stimulation, computational models and decoding algorithms: from signal processing to adaptive encoding, materials design and hardware implementation: from memristive synaptic devices to neuromorphic platforms, conclusions and future directions.

## Limitations of the study

This review synthesizes rapidly evolving work across neuroscience, neuromodulation, bioelectronic materials, and neuromorphic hardware. Because many memory-prosthetic studies remain small-cohort, preclinical, or technology-demonstration studies, the design rules proposed here should be interpreted as evidence-calibrated targets rather than validated clinical specifications. Long-term safety, durability, patient heterogeneity, algorithm adaptation, and comparative effectiveness across invasive and non-invasive approaches require larger longitudinal studies.

## Acknowledgments

This work was supported by the Leading Innovative and Entrepreneurial Team Program of Zhejiang, China (Grant No. 2023R01003), the 10.13039/501100002858China Postdoctoral Science Foundation, China (Grant Nos. 2025M783506 and 2026T190926), the 10.13039/501100004731Zhejiang Provincial Natural Science Foundation of China, China (Grant Nos. LQN25F010005 and LMS26F010011), and the 10.13039/501100001809National Natural Science Foundation of China, China (Grant No. 62501217).

## Author contributions

F.M.: conceptualization, supervision, project administration, writing – original draft, and writing – review and editing. X.Z.: conceptualization, visualization, and writing – review and editing. Y.X.: investigation, methodology, and writing – original draft. C.T.: investigation and writing – original draft. D.Z.: investigation, methodology, and writing – original draft. S.L.: investigation and writing – original draft. D.C.: investigation and writing – original draft. W.L.: investigation, visualization, and writing – original draft. Z.S.: conceptualization, supervision, funding acquisition, and writing – review and editing. S.H.: conceptualization, supervision, and writing – review and editing.

## Declaration of interests

The authors declare no competing interests.

## Declaration of generative AI and AI-assisted technologies in the writing process

During the preparation of this work, the authors used OpenAI’s ChatGPT/Codex for language editing, formatting support, and final-file checklist review. After using this tool/service, the authors reviewed and edited the content as needed and take full responsibility for the content of the publication.
